# Improving pharmacy practice through public health programs: experience from Global HIV/AIDS initiative Nigeria project

**DOI:** 10.1186/2193-1801-2-525

**Published:** 2013-10-17

**Authors:** Dorothy Oqua, Kenneth Anene Agu, Mohammed Alfa Isah, Obialunamma U Onoh, Paul G Iyaji, Anthony K Wutoh, Rosalyn C King

**Affiliations:** Howard University Pharmacists And Continuing Education (PACE) Center, Plot 1073 J. S. Tarka Street, Area 3, Garki PO BOX 10435, Abuja, Nigeria; Pharmacists And Continuing Education (PACE) Center, Howard University College of Pharmacy, Washington, DC USA

**Keywords:** Pharmaceutical care, HIV/AIDS, Public health programs, Patients, Nigeria

## Abstract

**Background:**

The use of medicines is an essential component of many public health programs (PHPs). Medicines are important not only for their capacity to treat and prevent diseases. The public confidence in healthcare system is inevitably linked to their confidence in the availability of safe and effective medicines and the measures for ensuring their rational use. However, pharmacy services component receives little or no attention in most public health programs in developing countries. This article describes the strategies, lessons learnt, and some accomplishments of Howard University Pharmacists and Continuing Education (HU-PACE) Centre towards improving hospital pharmacy practice through PHP in Nigeria.

**Method:**

In a cross-sectional survey, 60 hospital pharmacies were randomly selected from 184 GHAIN-supported health facilities. The assessment was conducted at baseline and repeated after at least 12 months post-intervention using a study-specific instrument. Interventions included engagement of stakeholders; provision of standards for infrastructural upgrade; development of curricula and modules for training of pharmacy personnel; provision of job aids and tools amongst others. A follow-up hands-on skill enhancement based on identified gaps was conducted. Chi-square was used for inferential statistics. All reported p-values were 2-tailed at 95% confidence interval.

**Results:**

The mean duration of service provision at post-intervention assessment was 24.39 (95% CI, 21.70–27.08) months. About 16.7% of pharmacies reported been trained in HIV care at pre-intervention compared to 83.3% at post-intervention. The proportion of pharmacies with audio-visual privacy for patient counseling increased significantly from 30.9% at pre-intervention to 81.4% at post-intervention. Filled prescriptions were cross-checked by pharmacist (61.9%) and pharmacy technician (23.8%) before dispensing at pre-intervention compared to pharmacist (93.1%) and pharmacy technician (6.9%) at post intervention. 40.0% of pharmacies reported tracking consumption of drugs at pre-intervention compared to 98.3% at post-intervention; while 81.7% of pharmacies reported performing periodic stock reconciliation at pre-intervention compared to 100.0% at post-intervention. 36.5% of pharmacies were observed providing individual counseling on medication use to patients at pre-intervention compared to 73.2% at post-intervention; and 11.7% of pharmacies had evidence of monitoring and reporting of suspected adverse drug reaction at pre-intervention compared to 73.3% at post-intervention. The institution of access to patients’ clinical information by pharmacists in all pharmacies at post-intervention was a paradigm shift.

**Conclusion:**

Through public health program, HU-PACE created an enabling environment and improved capacity of pharmacy personnel for quality HIV/AIDS and TB services. This has contributed in diverse ways to better monitoring of patients on pharmacotherapy by pharmacists through access of pharmacists to patients’ clinical information.

## Background

The scope of pharmacy practice includes more traditional product-oriented roles such as compounding, supply and dispensing medications, care and custody of drugs, drug tendering and purchasing, record keeping and accounting and administrative functions (Erah 2003). It now embraces more modern patient-focused and outcome-oriented pharmaceutical care (Erah 2003; Joda and Nwaokomah 2011). Pharmaceutical care is the responsible provision of medication-related care designed to achieve definite outcomes that improve a patient’s quality of life (Hepler and Strand 1990; King and Fomundam 2010). Pharmaceutical care practices involve identifying, resolving and preventing actual or potential drug-related problems.

Pharmacists in many developed countries are practically promoting pharmaceutical care as a philosophy and standard of provision of care for patients. Although pharmaceutical care has become a preferred mode of practice (Erah and Nwazuoke 2002; Farris et al. 2005) and the attitudes of pharmacists towards pharmaceutical care are favorably high irrespective of the practice settings (Swift 1993). However, it has not been fully integrated into professional pharmacy practice as a standard of care for patients in Nigeria (Erah and Nwazuoke 2002). In the tertiary health facilities with its full complement of skilled and competent staff, the provision of pharmaceutical care is still at a rudimentary stage. In most hospitals, pharmacists have no access to patient clinical information other than the prescription sheet for the day and all prescriptions are filled through the window system of dispensing with no opportunity for audiovisual privacy nor attempt to track the patient’s medication profile, provide medication adherence counseling, screen or monitor for adherence or adverse drug reactions, early warning signs of treatment failure and drug resistance.

Patient-oriented pharmacy practice in Nigerian hospitals is limited by inadequate infrastructure, lack of proper coordination of activities, resistance of physicians against patient-oriented pharmaceutical services especially in hospital wards, lack of proper training for pharmacists and lack of self-confidence (Erah and Nwazuoke 2002). Other barriers to pharmaceutical care implementation include not having enough time to talk with patients, pharmacist’s perception that patients are not willing to pay for this intensive level of care and poor staffing (Erah and Nwazuoke 2002; Louie and Robertson 1993; May 1993; World Health Organization 2010). According to the World Health Organization (WHO) Health Statistics report (2010), Nigeria has one pharmacist per 10,000 of the population (World Health Organization 2010; Joda and Nwaokomah 2011). Therefore, only 10 pharmacists are available for 100,000 people compared to minimum of 50:100,000 recommended due to the increasing demand for pharmacists in public health (Joda and Nwaokomah 2011; Oparah and Eferakeya 2005). However, there are no gold standards for assessing the adequacy of the health workforce to address the health care needs of a given population. Low density of health personnel usually suggests inadequate capacity to meet minimum coverage of essential services (World Health Organization 2012).

The pharmacy practice in Nigeria has remained focused largely on the products (drugs) even though many are aware of the changing role of the clinical pharmacist globally and the emphasis on patient-centered pharmaceutical care as a standard of care for patients especially those on chronic therapy. However, Nigerian pharmacists indicated willingness to implement pharmaceutical care but expressed major concerns about their knowledge, professional skills, and the pharmacy layout (Oparah and Eferakeya 2005).

Public health is the science or art of preventing disease, prolonging life and promoting health and efficiency through organized community effort (American Society of Health-System Pharmacists 2008). The roles of pharmacists in public health include but no limited to the following: population-based care; disease prevention and medication safety; health education including patient education programs on safe and effective medication use and other public health-related topics; public health policy development; research and training (American Society of Health-System Pharmacists 2008 American Society of Health-System Pharmacists 1997). The use of medicines is an essential component of many public health programs (PHPs) that are designed to improve the health of a target population. Medicines are important because of their capacity to treat and prevent diseases and to support PHPs. They are also important because the confidence of the public in the health policies of their countries is inevitably linked to their confidence in the availability of safe and effective medicines and the measures for ensuring their rational use. This requires a robust pharmacy services component as a fundamental part of all public health programs. Despite this fact, pharmacy services component receives little or no attention in most public health programs in most developing countries.

Based on this background the pharmacy services component of the Global HIV/AIDS Initiative Nigeria (GHAIN) project was proposed and led by Howard University through its Pharmacists and Continuing Education (HU-PACE) Centre. GHAIN is a public health interventions project aimed at reducing the impact of HIV/AIDS and tuberculosis in Nigeria through HIV prevention services, care and support services, and treatment services and interventions. The project was funded by the US President Emergency Plan for AIDS Relief (PEPFAR) through U.S Agency for International Development (USAID); and implemented by a consortium of four partners: HU PACE, AXIOS Foundation, German Leprosy Relief Association (GLRA) and the lead partner, FHI360. Prior to the GHAIN project, only a few of the estimated 3.5 million people living with HIV/AIDS in Nigeria had access to antiretroviral therapy (ART). They accessed treatment at private hospitals or procured their medication from community pharmacies that stocked them. In late 2001, the Government of Nigeria commenced the national HIV/AIDS treatment program in 25 tertiary facilities and in 2002 initiated the Prevention of Mother to Child Transmission (PMTCT) program in 11 tertiary facilities. GHAIN supported the provision of comprehensive HIV care and treatment services at secondary health facilities in the 36 states plus Federal Capital Territory of Nigeria. It supported 184 health facilities to provide prevention of mother-to-child transmission of HIV (PMTCT) services; 842,240 pregnant women were reached with HIV counseling and testing received their test result; while 36,520 HIV-positive pregnant women were provided prophylaxis with antiretroviral drugs (GHAIN M&E Monthly Bulletin 2011). It also supported 125 comprehensive ART sites; ever provided antiretroviral therapy (ART) to 161,120 HIV-positive patients, of which 124,596 of them were currently receiving ART as at the end of February 2011 (GHAIN M&E Monthly Bulletin 2011).

HU-PACE mandate in GHAIN was to strengthen pharmacy systems and services at health facilities and in the community by expanding pharmacists’ capacity to provide pharmaceutical care for those infected with HIV/AIDS, STI, and TB; and their families and by ensuring quality of care and drugs inventory control at the facility level in the target states in Nigeria. The pharmacy services component of the project should take a holistic view on the elements of pharmacy best practices and the pharmacy system in relation to the target health problems (HIV/AIDS and Tuberculosis). These elements include dispensing, patient counselling, refilling, patient adherence, referral process, education programs, interaction with other health team, data production and collection, and control of drugs. Baseline assessment indicated that most health facilities had inadequate pharmacy personnel and did not have the skills and competencies to manage clients on HIV treatment. While a constant supply of drugs could be obtained with an effective supply chain management system, optimum therapeutic outcomes for HIV clients initiated on therapy could only be assured by the responsible provision of patient focused pharmaceutical services. The pharmacy personnel needed to possess the requisite skills and information in a good pharmacy layout for a paradigm shift of pharmaceutical care as a theoretical statement to a professional practice in Nigeria. This article describes the HU PACE strategies, activities and some accomplishments towards improving hospital pharmacy practice in Nigeria.

## Methods

### Study design

This involved both cross-sectional and interventional study designs. Cross sectional surveys of selected hospital pharmacies was conducted at baseline (pre-intervention) and then repeated after at least 12 months post-intervention. The baseline assessment of the pharmacy systems and services was conducted to identify site specific gaps for targeted interventions.

### Settings

The study setting included primary, secondary and tertiary health facilities in Nigeria. There are three levels of public health care in Nigeria – primary, secondary and tertiary, which are owned and managed by Local Government Authorities, State Government and Federal Government of Nigeria respectively. These 3 levels of health care are connected through a referral system. The GHAIN project supported secondary and tertiary health facilities to offer comprehensive HIV care services including both antiretroviral therapy (ART) and prevention of mother to child transmission of HIV (PMTCT) or just PMTCT stand-alone services. The primary health facilities were supported to provide mainly PMTCT stand-alone services. All the health facilities have a distinct pharmacy department responsible for providing pharmaceutical services to the patients.

### Population and sample

The population for the study sites included 184 health facilities supported by GHAIN project spreading across all 36 states plus Federal Capital Territory of Nigeria. Sixty health facilities were selected from this population for the post-intervention assessment using simple random sampling technique.

### Inclusion/exclusion criteria

All health facilities that had record of the pre-intervention assessment of the hospital pharmacies, provided HIV care services to HIV-infected persons for ≥12 months’ duration of post-intervention and supported by GHAIN project were included in this assessment. All health facilities that which did not meet these criteria were excluded from the study.

### Interventions

HU-PACE strategies for achieving its mandate in GHAIN project involved sensitization and mobilization of relevant members of the Pharmaceutical Society of Nigeria (PSN), the Pharmacists Council of Nigeria (PCN), the National Institute for Pharmaceutical Research and Development (NIPRD), the National Agency for Food and Drug Control (NAFDAC) and the Food & Drugs Division of Federal Ministry of Health through a launch of the pharmacy component of GHAIN.

Other interventions included the provision of standards for infrastructural upgrade of the pharmacy environment such as the renovations of counselling rooms to guarantee audio-visual privacy; provision of standards for appropriate drug storage such as lockable cupboards, shelves and pallets, refrigerators, wall and fridge thermometers and functional air conditioning. The interventions also included the development of a curriculum and modules for a training of pharmacists and lower cadre pharmacy personnel as needed; development and provision of required job aids and tools, and conduct of centralized didactic trainings prior to the activation of facilities for service provision.

A follow-up onsite skill enhancement to ensure application of knowledge and skills in service provision and accurate documentation was conducted at the inception of HIV comprehensive services through a hands-on training of the pharmacy staff on selected elements of pharmacy best practices using Pharmacy State Coordinators who are recognized leaders in the Nigerian pharmacy community. This role was later transitioned to State Directors of Pharmaceutical Services (DPS) who are responsible for maintaining the standards of pharmacy practice in Nigeria. Training modules developed for pharmacists included all components of HIV pharmaceutical care, clinical pharmacovigilance of ARV drugs, and pharmacy best practices elements focusing on dispensing, patient counselling, refilling, patient adherence, referral process, education programs, interaction with other health team members, data production and collection and control of drugs. In addition, a level adjusted curriculum focusing on drug dispensing and documentation for HIV/AIDS services was developed for lower cadre pharmacy and support personnel within primary, secondary and tertiary level of care.

Tools developed for documentation of pharmaceutical care services and for drug inventory control included the pharmacy order form for prescription ordering, pharmaceutical care daily worksheet for daily documentation of pharmaceutical care provided to clients including the screening and documentation of drug therapy problems and the interventions in the pharmacy; pharmacy daily worksheet for daily documentation of prescription orders filled in the pharmacy; monthly work books, and monthly summary forms, pharmacy appointment diaries, and patient status registers. The National Individual Case Safety Report Form was deployed for reporting of suspected ADRs to the National Pharmacovigilance center, National Agency for Food, Drug Administration and Control (NAFDAC). Some IEC materials and job aids provided were dispensing trays, auxiliary medication labels with label key charts, key to grading adverse drug reactions, plain medication labels, pharmacy jackets, and pharmacists’ tags.

To overcome the acute shortage of pharmacists at the project pharmacies amidst increasing patient load and associated documentation, GHAIN conceptualised and set up the HU-PACE Pharmacists Volunteer Scheme (HPVS). The HPVS provides trained volunteer hospital and community pharmacists as added resource to support hospitals with high patient load to provide HIV related pharmaceutical services at their convenience while serving as a pool of skilled pharmacists who can provide HIV pharmaceutical care at their primary places of practice.

Sustainability strategies that was implemented include the training of all states government employed Directors of Pharmaceutical Services who have the primary responsibility for providing oversight to these hospital pharmacies. They were trained on Pharmaceutical care in HIV/AIDS and Pharmacy Best Practice elements to build their capacity in providing technical supervision. They were also involved in the hands-on pharmacy best practices training of health facility staff, joint monitoring and supportive supervisory visits with project personnel and the conduct of bimonthly peer-review and feedback meetings. The pharmaceutical care data generated from all supported facilities in each state were also reviewed for any emerging trends at this meeting. The project also collaborated with the Pharmacist Council of Nigeria to incorporate project training modules into the curriculum for mandatory continuing professional development for pharmacists in Nigeria. In collaboration with NAFDAC and the National Drug Safety Advisory Committee, resource materials on Clinical Pharmacovigilance of ARV drugs was developed and disseminated.

### Service package for HIV-positive clients

Following the interventions, the HU PACE supported service package for HIV-positive clients at the health facilities included routine medication adherence counselling for pre-ART, ART, and PMTCT patients; screening and reporting of medication errors; screening and reporting of adverse drug reactions; resolution of potential or actual drug therapy problems; and drug inventory management. Patient-focused dispensing information covers the medication use information, drug regimen, adherence, drug storage, possible interactions, adverse effects and cautionary measures amongst others.

### Data collection

The data collection was done using a project-specific pharmacy interview guide. The key sections of the guide included human resources and staff capacity, interaction between pharmacist and clients, infrastructure, availability of drugs, logistics, guidelines and procedures. The interview guide was used by the researchers and the trained assistants to elicit responses from the participants on the questions items. Other methods used for data collection included direct observations and review of records. Management approval was obtained from the management of the hospitals. Ethical exemption was obtained from Health Research Ethics Committee, Abuja Nigeria. Informed consent was also obtained from the participants. The respondents consisted of facility pharmacists or a pharmacy staff who is very knowledgeable about the project pharmacies.

### Monitoring and evaluation

Continuous quality improvement was ensured by Monitoring and Evaluation Pharmacists through routine monitoring and supportive supervision using pharmacy practice log, service quality assessment checklist and periodic data verification and collation in the pharmacy. In addition, GHAIN initiated periodic performance review and feedback meetings of stakeholders led by State Directors of Pharmaceutical Services (DPS), to jointly address quality related issues. Quality improvement measures were implemented throughout the life of the project and included retraining as appropriate, to provide updates and share new concepts, providing support to initiate or strengthen existing Medicine and Therapeutic committees and ARVs Pharmacovigilance sub-committees within the hospitals.

The Predictive Analytical SoftWare (PASW®) was used for data analysis. Descriptive statistics was used to describe the key variables of interest. Chi-square was used for inferential statistics. All reported p-values were 2-tailed at 95% confidence interval.

## Results

GHAIN through HU-PACE has trained and retrained 7563 of various categories of pharmacy personnel including pharmacists and pharmacy technicians over the life of the project. HU-PACE trained hospital pharmacists, volunteer pharmacists and pharmacy support personnel have provided clinical pharmaceutical services to over 160,000 HIV infected clients (including 9,499 children) receiving antiretroviral treatment (ART) of which majority are currently retained on therapy. Pharmaceutical care has also been provided to over 36,000 HIV infected pregnant women receiving ARV prophylaxis for PMTCT; 2,857 clients receiving occupational and non-occupational post exposure ARV prophylaxis; 495,565 clients receiving drugs and other clinical care for management of opportunistic infections; and 159,401 clients receiving Co-trimoxazole prophylaxis. The access to patients’ clinical information by pharmacists was instituted in all supported health facilities.

The collaboration with the Pharmacists Council of Nigeria (PCN) led to the inclusion of the GHAIN training modules for Mandatory Continuous Pharmacy Development in Nigeria. Some of the tools, job aids and other resource materials developed, produced and disseminated include Best Practice Guide for Project Pharmacies in Health Facilities, Participant’s Manual for Pharmacovigilance for Antiretroviral Drugs Training for Health Care Professionals, Participant’s Manual for Pharmaceutical Care in HIV/AIDS Training for Hospital Pharmacists, Medication Dispensing Process poster, Quick Reference Guide on Drugs for HIV/AIDS Management, and standard operating procedures (SOPS) for project pharmacies.

In Cross River State of Nigeria, a public private partnership evolved with trained private pharmacists providing professional services at hospital pharmacies without resident pharmacists and at some primary health care centres based on memorandum of understanding (MOU) under the State universal access program.

The mean duration of service provision at post-intervention assessment was 24.39 (95% CI, 21.70–27.08) months. The 60 health facilities comprised of 9 (15.0%) primary health facilities, 49 (81.7%) secondary health facilities and 2 (3.3%) tertiary health facilities. Of the 60 sites for assessment, 63.3% were offering comprehensive ART and PMTCT services while 36.7% were providing only PMTCT services.

### Human resources and staff capacity

The total number of staff members providing services in the pharmacy at these facilities changed by 30.2% over the observation period (Table 1). This increase did not improve the inadequate human resources situation at these facilities significantly (p = 0.077). At pre-intervention assessment, 75.0% of the facilities reported to have at least one pharmacist providing services in the pharmacy (of which only 5.1% were on part-time equivalent) compared to 93.3% at post-intervention assessment (of which 18.9% were on part-time equivalent). This represents 18.3% increase in this category of human resources in pharmacy which was also not statistical significant (p = 0.065).

**Table 1:** **Frequency distribution of human resources and staff capacity in pharmacy of the facilities (n = 60)**

Pharmacy human resources	Frequency (Percentage)	
	Pre -intervention	Post-intervention
Pharmacists	158 (36.7)	233 (41.5)
Pharmacy technicians	104 (24.1)	139 (24.8)
Pharmacy attendants and other staff	169 (39.2)	189 (33.7)
**Type of training**		
Rational use of ARVs	1 (1.7)	0 (0.0)
HIV comprehensive care	3 (5.0)	5 (8.3)
PMTCT training	5 (8.3)	15 (25.0)
Pharmaceutical care in HIV training	2 (3.3)	28 (46.7)
Pharmacy best practice training	1 (1.7)	28 (46.7)
Logistics Management Information System for ARVs	2 (3.3)	9 (15.0)
Antiretroviral Therapy (ART) Training	0 (0.0)	13 (21.7)
ART Standard Operating Procedures and Adherence	0 (0.0)	15 (25.0)
ARV dispensing & documentation	0 (0.0)	1 (1.7)
Ready-to-Use Therapeutic Food (RUTF) for the treatment of pediatric Severe Acute Malnutrition (SAM)	0 (0.0)	2 (3.3)
Electronic Medical Record (LAMIS) Training	0 (0.0)	1 (1.7)
HIV Post-Exposure Prophylaxis (PEP)	5 (8.3)	42 (70.0)

Of the 60 health facilities, 10 (16.7%) reported in the least that the pharmacy staff received training in HIV care at pre-intervention assessment compared to 50 (83.3%) at post-intervention assessment. The two most frequent trainings reported were Pharmaceutical Care in HIV and Pharmacy Best Practice Trainings at post-intervention assessment (Table 1).

### Interaction between pharmacist and clients

**Figure 1:**
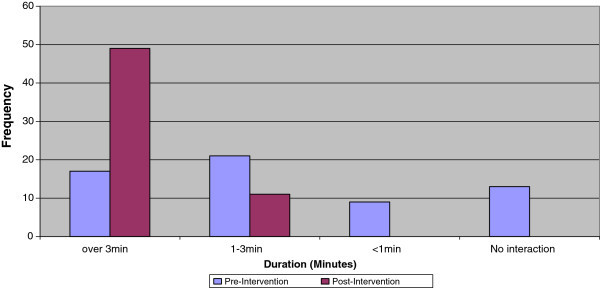
**Analysis of average length of interpersonal interactions between the Pharmacists and the Patient after intervention (n=60 sites).**

**Figure 2:**
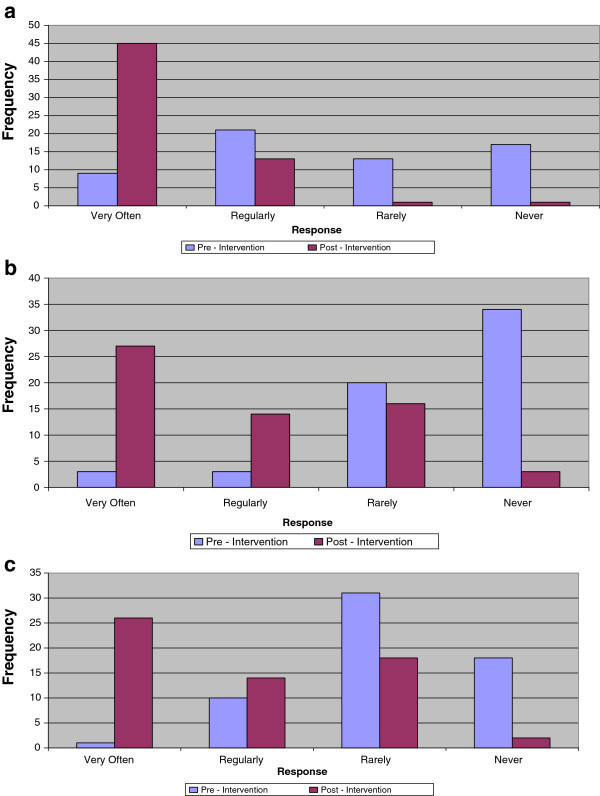
**Comparison of the types of information shared during Pharmacist-Patient interactions (n = 60). a)** Detailed instruction on taking medications. **b)** Other HIV/AIDS prevention messages. **c)** General nutrition and well-being.

Analysis of the interpersonal interaction between pharmacist and clients showed that 81.7% of the interactions lasted for over 3 minutes post-intervention compared to 28.3% at pre-intervention (Figure 1). The provision of information related to medication use, other HIV/AIDS prevention messages, general nutrition and well-being of patients occurred less frequently during the pharmacist-patient interpersonal interactions at pre-intervention compared with the findings from post-intervention assessment (Figures 2a–c).

The percentage of hospital pharmacies that reported having IEC materials to support client-provider interaction increased from 20.0% at pre-intervention to 68.3% at post-intervention assessments. The number of hospital pharmacies with IEC materials related to medication use information increased from 7 (11.7%) at pre-intervention to 31 (51.7%) at post-intervention; specific disease information increased from 5 (8.3%) at pre-intervention to 35 (58.3%) at post-intervention; Healthier lifestyle or Nutrition information increased from 6 (10%) at pre-intervention to 30 (50.0%) at post-intervention; HIV/AIDS Prevention/ treatment information increased from 5 (8.3%) at pre-intervention to 33 (55.0%) at post-intervention; Safer Sex Information increased from 7 (11.7%) at pre-intervention to 30 (50.0%) at post-intervention.

### Infrastructure

The percentage of hospital pharmacies with audio-visual privacy in the patient counseling and dispensing area increased significantly from 30.9% at pre-intervention to 81.4% at post-intervention assessment. Those that had neither visual nor auditory privacy decreased from 69.1% at pre-intervention to 15.3% at post intervention. None of the hospital pharmacies had either visual or audio privacy at pre-intervention compared to 1.7% at post intervention. An improvement of over 50% was reported and observed regarding the presence of pharmacy reference books/materials at post-intervention assessment (86.2%) compared to 42.6% reported at pre-intervention.

### Logistics

Filled prescriptions were cross-checked by pharmacist (61.9%) and pharmacy technician (23.8%) before they are given to patients or caregivers at pre-intervention compared to pharmacist (93.1%) and pharmacy technician (6.9%) at post intervention. Forty percent (40.0%) of hospital pharmacies reported tracking consumption of drugs using dispensing records at pre-intervention compared to 98.3% hospital pharmacies at post-intervention assessment.

At pre-intervention, 49 (81.7%) hospital pharmacies reported performing periodic stock reconciliation by comparing stock on hand with inventory records (of which 0.0% were daily, 18.4% weekly, 0.0% biweekly, 38.8% monthly, 2.0% bimonthly, 34.7% quarterly, and 4.1% were semi-annually). At post-intervention, 60 (100.0%) hospital pharmacies reported performing periodic stock reconciliation (of which 1.7% were daily, 16.7% weekly, 1.7% biweekly, 73.3% monthly, 3.3% bimonthly, 1.7% quarterly and 0.0% were semi-annually). Of the hospital pharmacies, 42 (70.0%) reported generating periodic stocks reports at pre-intervention compared to 60 (100.0%) at post-intervention assessment. The average turn-around time between placing an order and receiving the supplies at the pharmacy changed from 39.5% two weeks, 31.6% one month, and 28.9% two months at pre-intervention to 44.6% two weeks, 46.4% one month, and 8.9% two months at post-intervention.

### Guidelines and procedures

**Figure 3:**
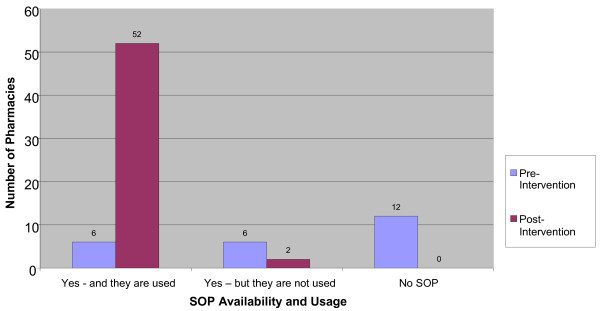
**Availability and usage of standard operating procedure (SOP) for Pharmacy service (n=60).**

The standard operating procedures (SOP) for pharmacy services were unavailable and not used in majority of the hospital pharmacies at pre-intervention as opposed to the findings at post-intervention (Figure 3).

The percentage of hospital pharmacies whose pharmacists were observed providing individual counseling to patients on medication use increased from 36.5% at pre-intervention to 73.2% at post-intervention. The proportion of hospital pharmacies with evidence of monitoring and reporting of suspected adverse drug reaction (ADR) increased from 7 (11.7%) at pre-intervention to 44 (73.3%) at post-intervention assessment.

Documented procedures for monitoring and reporting of medication errors were observed in 5 (8.3%) hospital pharmacies at pre-intervention compared to 37 (61.7%) pharmacies at post-intervention assessment. However, the medication errors reporting form was observed in only 2 (3.3%) of the hospital pharmacies at pre-intervention compared to 44 (73.3%) pharmacies at post-intervention.

## Discussion

Some of the gaps identified at pre-intervention assessment included inadequate pharmacy personnel, lack/inadequate skills and competencies to manage clients on HIV treatment in most facilities. The patient counseling and dispensing area had no audio-visual privacy for clients and the duration of interpersonal interaction between pharmacist and clients in most facilities were <3 minutes and mainly through a window. Evidence of patient-focused care, monitoring of drug therapy problems including adverse drug reactions, medication errors, medication adherence and the interventions were not existent in most facilities. These identified gaps are consistent with factors that limited patient-oriented pharmacy practice in Nigerian hospitals in previous reports (Erah and Nwazuoke 2002; Louie and Robertson 1993; May 1993; World Health Organization 2010).

GHAIN project expanded access of thousands of HIV-infected clients (adults and children) to antiretroviral therapy (ART) and HIV-infected pregnant women to antiretroviral drug prophylaxis across all states of Nigeria compared to a few in the 2001 and 2002. The involvement of pharmacists in HIV prevention, treatment, care and support services was very limited or non-existent before the GHAIN programme in Nigeria. Many public health programmes in Nigeria did not see the role of pharmacists beyond the pharmaceutical management of drugs for accountability and the maintenance of an efficient supply chain and storage system for drugs and other commodities. This is changing now and GHAIN programme pioneered this paradigm shift with the distinct pharmacy-based interventions of the programme.

With the supported infrastructural upgrades in hospital pharmacies, audio-visual privacy was entrenched in most health facilities to provide good patient counseling environment. This facilitated confidentiality, phased out dispensing of medicines through the window and ensured better interpersonal pharmacist-patient relationship which is essential in chronic therapy. Consequently, the patient-pharmacist interpersonal contact time improved significantly. HU-PACE expanded the skills of hospital pharmacists to provide patient-centered pharmaceutical care to thousands of HIV-infected adults and children including pregnant women for optimal outcome of pharmacotherapy. Better monitoring and counselling of patients on antiretroviral therapy or prophylaxis by pharmacists was instituted through capacity building and access to patients’ clinical information in all supported hospital pharmacies to prevent potential or actual drug related problems.

HU-PACE installed the practice of pharmaceutical care for HIV infected clients accessing services within pharmacies in primary, secondary and tertiary health facilities with appropriate documentation of services provided. The quality of pharmaceutical care provided to HIV clients improved through the years. Routine screening of clients on ARV drugs for adverse drug reactions and its reporting has become institutionalised in all hospital pharmacies through the use of pharmaceutical care daily worksheet. This did not exist at pre-intervention assessment.

The inclusion of the GHAIN training modules into the curriculum for Mandatory Continuous Pharmacy Development by Pharmacists Council of Nigeria (PCN) could ensure the sustainability of the training interventions. HU-PACE Pharmacists Volunteer Scheme (HPVS) instituted to mitigate human resources gap at the hospital pharmacies was instrumental to the maintenance of the provision of pharmaceutical care at the health facilities. However, this did not improve the inadequate human resources for pharmacy significantly. The scheme resulted to a public-private partnership between Cross River State of Nigeria and trained private pharmacists who provided professional services at hospital pharmacies without resident pharmacists and at some primary health care centres based on memorandum of understanding (MOU) under the State universal access program.

### Challenges

One of the challenges faced during project implementation was the paucity of pharmacists in many health facilities. There was a lack of trainable pharmacy personnel in some primary health care facilities. There were complaints from trained personnel in supported facilities about increased workload and the associated documentation as more clients were enrolled on therapy. There was frequent transfer-out of trained staff to non-supported hospitals. This periodic transfer-out of staff is inadvertently an integral part of the public service rules in the States Ministry of Health. This problem may be improved through increased pre-service training, increased employment of appropriate personnel as well as the strategic posting of skilled and competent pharmacy personnel who can provide the required professional and qualitative services to HIV clients on therapy. There was limited commitment to programme ownership from government personnel which may have compromised programme sustainability.

### Lessons learnt

Volunteerism could be a potent force for resolving the human resource issues in pharmacies. The GHAIN pharmacy volunteer scheme and the lower-cadre site health workers were invaluable in addressing manpower shortages in the health system.

Pharmaceutical care interventions can improve the reporting of adverse drug reactions in HIV treatment centers. The involvement of senior pharmacy personnel as stakeholders encouraged ownership at the highest level. Public health programmes can serve as a platform for improving pharmacy practice in Nigeria.

## Conclusion

Through public health program, HU-PACE created an enabling environment and improved capacity of pharmacy personnel for quality HIV/AIDS and TB services. This has contributed in diverse ways to better monitoring of patients on pharmacotherapy by pharmacists through access of pharmacists to patients’ clinical information. This was threatened by inadequate human resources for health at the pharmacies but was mitigated to a reasonable extent by the HU-PACE Pharmacists Volunteer Scheme (HPVS).
